# Prognosis and Outcome of Carbapenem-Resistant Enterobacterales Bacteremia Managed With Ceftazidime-Avibactam and Aztreonam Combination Therapy in Tawam Hospital, UAE: A Retrospective Study

**DOI:** 10.7759/cureus.85689

**Published:** 2025-06-10

**Authors:** Abla Agha, Ali Al Hassani, Aya Shubbar, Zaid Al Hassani, Ahmed Al Hassani, Aqeel Saleem

**Affiliations:** 1 Infectious Diseases, Sheikh Tahnoon Bin Mohammed Medical City, Al Ain, ARE; 2 Internal Medicine, Tawam Hospital, Al Ain, ARE; 3 College of Medicine, University of Sharjah, Sharjah, ARE

**Keywords:** aztreonam, bacteremia, carbapenem-resistant enterobacterales (cre), ceftazidime-avibactam, tawam hospital

## Abstract

Introduction

In recent years, the medical community has grown increasingly alarmed by the escalating rates of carbapenem resistance - a global concern that is also affecting the United Arab Emirates (UAE). This rise in antibiotic resistance poses a significant challenge to healthcare systems and necessitates urgent and comprehensive research. The primary objective of this study is to investigate the factors that influence the prognosis and outcomes of bacteremia caused by carbapenem-resistant Enterobacterales (CRE), managed with a combination of ceftazidime-avibactam (CAZ-AVI) and aztreonam (ATM). Understanding the determinants of treatment success may provide valuable insights into improving patient care and outcomes.

Methods

This retrospective observational chart review was conducted at Tawam Hospital, Al Ain, from 2020 to 2023. Seventeen adult patients (aged >18 years) with confirmed CRE bacteremia who received combination therapy with CAZ-AVI and ATM were included. Data were extracted from the SEHA electronic medical records, including demographics, clinical features, laboratory findings, and outcomes such as ICU admission, in-hospital mortality, and length of stay. Statistical analyses were performed using Excel, Meta-Chart, and SkyBlue Statistics. Given the small sample size, descriptive statistics were prioritized, and chi-square and unpaired t-tests were used to explore associations, recognizing limitations in statistical power.

Results

The incidence of CRE bacteremia treated with CAZ-AVI and ATM increased over the study period, with the highest number of cases recorded in 2023. Antimicrobial resistance remained consistently high across both beta-lactam and non-beta-lactam classes. The overall in-hospital mortality rate was 29.4%, with long-term four-year mortality reaching 53%. The median length of hospital stay was 19 days, and 17.6% of patients required intensive care. Poor outcomes were primarily associated with immunosuppression, prior hospitalizations, and multiple comorbidities.

Conclusion

This study highlights the increasing clinical burden of CRE bacteremia in the UAE. By identifying key prognostic factors and reporting high mortality and prolonged hospital stays despite combination therapy, it underscores the urgent need for timely intervention, improved antimicrobial stewardship, and enhanced diagnostic capacity. These findings contribute valuable regional data to the global effort to curb antimicrobial resistance.

## Introduction

Carbapenem-resistant Enterobacterales (CRE) have emerged as a significant global public health threat, associated with high morbidity, mortality, and limited treatment options. The World Health Organization (WHO) has classified CRE as a priority pathogen due to its resistance to last-line antibiotics, particularly carbapenems, which are often reserved for managing multidrug-resistant Gram-negative infections [1]. In the United Arab Emirates (UAE), the incidence of CRE infections has also increased, reflecting global trends and posing a growing challenge to healthcare systems in the region [2]. Alarmingly, projections indicate that by 2050, deaths attributable to antimicrobial resistance (AMR) may rival those caused by cancer [3].

Clinicians worldwide, including those in the UAE, increasingly face the challenge of managing carbapenem-resistant infections. The rising prevalence of CRE is particularly concerning given that carbapenems are reserved for severe infections unresponsive to other antimicrobial therapies [4]. The goal of this research is to identify factors influencing treatment outcomes and to assess the incidence and severity of CRE infections. Such insights will help clarify the magnitude of the problem and guide the evaluation of emerging therapeutic strategies.

CRE infections are associated with serious clinical consequences, including higher mortality rates, prolonged intensive care unit (ICU) stay, extended hospital stays, and substantial financial burdens on healthcare systems [5]. Studies have shown that CRE infections are associated with mortality rates ranging from 26% to 44%, prolong ICU stays by four to 14 days, extend hospital stays by four to 21 days, and impose significant financial burdens on healthcare systems [5,6]. Moreover, CRE organisms continue to evolve dynamic resistance profiles, further narrowing therapeutic options [7]. Therefore, continuous surveillance and resistance profiling are essential to optimize antibiotic use and clinical management [8].

Among emerging options, the combination of ceftazidime-avibactam (CAZ-AVI) and aztreonam (ATM) has shown promise in overcoming resistance mediated by metallo-beta-lactamases (MBLs). CAZ-AVI alone is ineffective against MBLs, while ATM is inherently stable against MBL hydrolysis but vulnerable to degradation by co-produced extended-spectrum beta-lactamases (ESBLs) or AmpC enzymes. When administered together, CAZ-AVI inhibits these coexisting enzymes, effectively protecting ATM and restoring its activity against MBL-producing organisms [6]. This pharmacological synergy forms the rationale for using this combination in settings where MBLs are prevalent and therapeutic alternatives are scarce.

Despite growing international interest in this approach, there is a notable lack of real-world data from the Middle East and the Gulf region evaluating clinical outcomes associated with CAZ-AVI and ATM combination therapy. Most published evidence arises from studies conducted in Europe or North America, where resistance mechanisms, prescribing practices, and healthcare infrastructure differ from those in the UAE [2,6]. This regional knowledge gap limits data-driven antimicrobial decision-making and highlights the need for context-specific research.

Accordingly, the objective of this study is to investigate the prognosis and clinical outcomes of CRE bacteremia treated with CAZ-AVI and ATM at Tawam Hospital, UAE, between 2020 and 2023. By analyzing prognostic factors and treatment outcomes, this research aims to generate actionable data to support local antimicrobial stewardship and enhance treatment strategies against multidrug-resistant pathogens.

## Materials and methods

Study design

This study was a retrospective observational chart review conducted over a four-year period from January 2020 to December 2023.

Study population and setting

This study included all adult patients (aged ≥18 years) admitted to Tawam Hospital between January 2020 and December 2023 who had confirmed bloodstream infections with CRE and were treated with a combination of CAZ-AVI and ATM. Patients were excluded if they were under 18 years of age, had CRE isolated only from non-blood culture sources (e.g., urine, sputum), did not receive combination therapy (e.g., monotherapy or sequential antibiotic use), or had incomplete medical records precluding outcome analysis. As this was a retrospective study, no formal sample size calculation was performed. The final cohort of 17 patients reflects all eligible cases identified within the study period. While the sample size is limited, it provides meaningful real-world insight into the clinical application of this novel antibiotic combination in a high-risk patient population.

**Figure 1:**
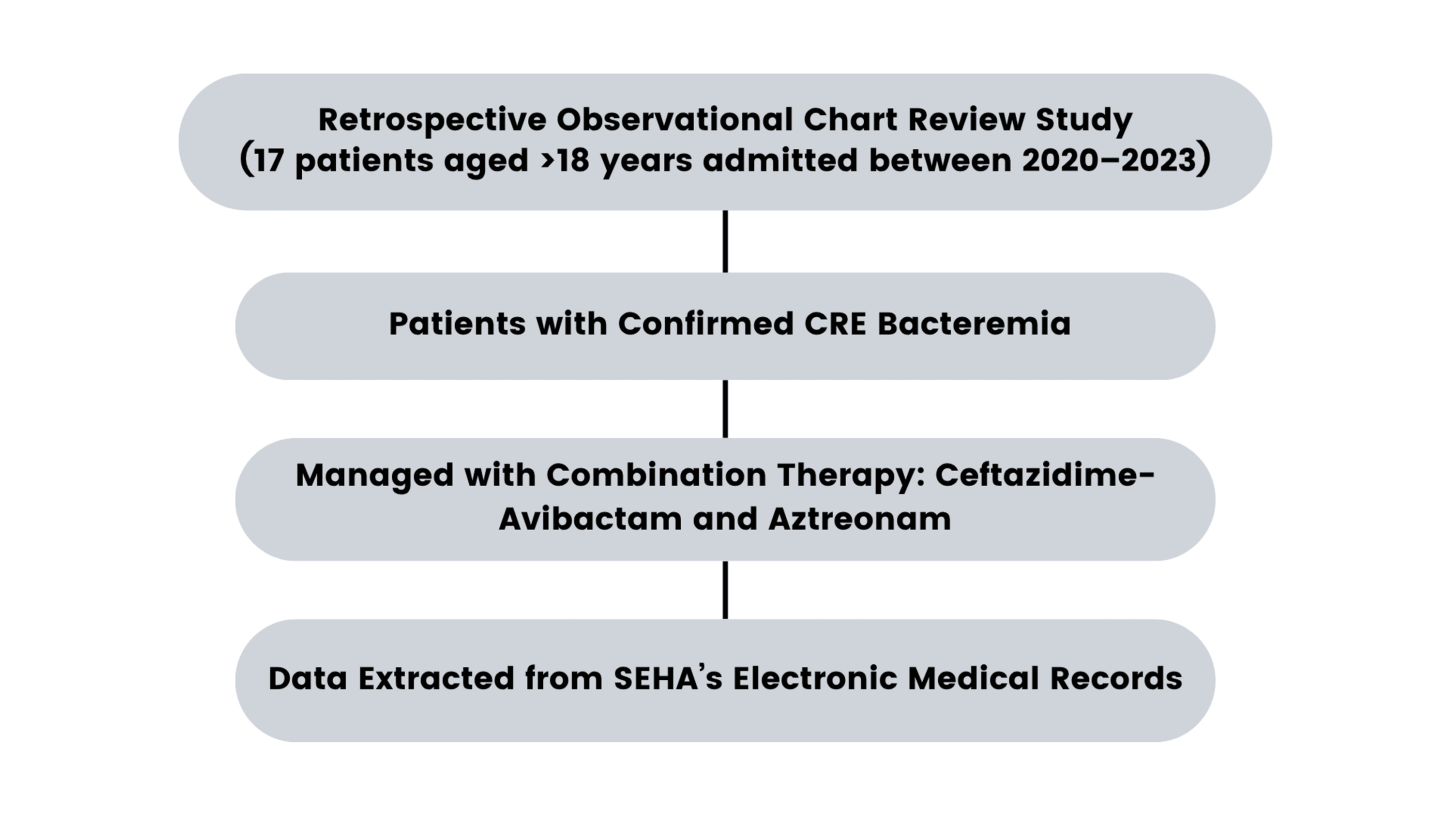
Study design overview for the retrospective observational analysis of CRE bacteremia managed with ceftazidime-avibactam and aztreonam at Tawam Hospital (2020–2023)

Study procedures and data source

Data were collected retrospectively from the SEHA electronic medical records system. Patient charts were reviewed to extract demographic information, clinical characteristics, microbiological findings, treatment details, and outcomes. All extracted data were de-identified to maintain patient confidentiality.

Measurements and variables

Quantitative variables collected included patient age, vital signs (temperature, blood pressure, heart rate, respiratory rate), complete blood count (CBC) results, and microbiological data from blood cultures, including antibiotic sensitivity profiles.

Categorical variables included comorbidities such as diabetes mellitus (DM), hypertension (HTN), chronic kidney disease (CKD), immunosuppressive status (defined as use of corticosteroids, chemotherapy, or biologics within 30 days), and active malignancy. These variables were assessed for their potential impact on infection severity and outcomes.

Outcome variables measured included length of hospital stay, ICU admission, in-hospital mortality, source of infection (hospital-acquired versus community-acquired), duration of antibiotic therapy, and occurrence of nosocomial complications.

Microbiological testing

Synergy testing between CAZ-AVI and ATM was performed using a modified E-test method. This involved applying two E-strips with overlapping gradients on Mueller-Hinton agar plates inoculated with the CRE isolate. Validation was based on in-house reproducibility and previous published protocols demonstrating correlation with broth microdilution methods. Results were interpreted based on the presence of a defined ellipse intersection, indicating synergy.

Bias consideration

Selection bias was minimized by including all eligible patients during the study period without sampling restrictions. Measurement bias was reduced by using standardized data extraction from the SEHA electronic medical records and relying on objective clinical and microbiological parameters. Two authors independently reviewed the data to enhance accuracy. While confounding could not be fully controlled, key variables such as ICU admission and comorbidities were analyzed to assess their potential impact on outcomes.

Statistical analysis

The sample size consisted of 17 patients who met the inclusion criteria. Data analysis was conducted using BlueSky Statistics software (BlueSky Statistics LLC, Chicago, USA). Descriptive statistics were used to summarize demographic, clinical, microbiological, and outcome variables. Continuous variables were reported as means and standard deviations, while categorical variables were expressed as frequencies and percentages. Group comparisons were performed using unpaired t-tests for continuous variables and chi-square tests for categorical variables. A p-value of <0.05 was considered statistically significant. Given the small sample size and retrospective design, the analyses were exploratory and interpreted with caution.

Ethical considerations

The study protocol was reviewed and approved by the Tawam Human Research Ethics Committee (ref. no. KD/AJ/1035) in accordance with the Declaration of Helsinki. Since the study involved a retrospective review of medical records and all data were de-identified, individual patient consent was waived. No identifiable personal information was collected, and patient confidentiality was strictly maintained throughout the study.

## Results

Due to the absence of molecular testing for carbapenemase genes at the facility, microbiological methods were employed to evaluate antimicrobial resistance patterns. Synergy between ATM and CAZ-AVI was assessed using a modified E-test-disc diffusion method. A CAZ-AVI E-test strip and an ATM disc (30 µg) were placed approximately 15 mm apart on Muller-Hinton agar plates inoculated with a 0.5 McFarland bacterial suspension. Plates were incubated at 37°C for 16 to 18 hours. Synergy was interpreted both qualitatively, by observing the presence of an inverse-D inhibition pattern, and quantitatively, by measuring inhibition zones according to Clinical and Laboratory Standards Institute (CLSI M100) breakpoints (Table 1). The minimum inhibitory concentration (MIC) breakpoints used were ≤4 µg/mL for ATM and ≤8 µg/mL for CAZ-AVI. This modified E-test method provided a practical approach for detecting synergy in the absence of advanced molecular diagnostics.

**Table 1:** Minimum inhibitory concentration (MIC) and disk diffusion breakpoints used for testing ceftazidime-avibactam and aztreonam synergy in carbapenem-resistant Enterobacterales (CRE) isolates

Organism	Antimicrobial	MIC breakpoints (μg/ml)			Disk breakpoints*^a^* (mm)		
		S	I*^b^*	R	S	I*^b^*	R
Enterobacterales	AZT	≤4	8^	≥16	≥21	18–20^	≤17
	CZA	≤8		≥16	≥21		≤20

Regarding antimicrobial dosing, CAZ-AVI was administered as an intravenous infusion at a standard dose of 2.5 g every eight hours, infused over two hours. Renal dose adjustments were applied as follows: no adjustment was required for patients with creatinine clearance (CrCl) >50 mL/min; for CrCl between 31 and 50 mL/min, the dose was reduced to 1.25 g IV every eight hours; for CrCl between 16 and 30 mL/min, a dose of 0.94 g IV every 12 hours was used; for CrCl between 6 and 15 mL/min, the dose was 0.94 g IV every 24 hours; and for CrCl <5 mL/min, the same dose was administered every 48 hours. In patients undergoing hemodialysis, CAZ-AVI was administered post-dialysis.

Aztreonam was administered concurrently as an intravenous infusion of 2 g every eight hours, with renal adjustments applied based on renal function. For patients with CrCl between 10 and 30 mL/min, a loading dose of 1-2 g was given, followed by 50% of the usual maintenance dose. For patients with CrCl <10 mL/min, a loading dose of 1-2 g was administered, followed by 25% of the maintenance dose. The two antibiotics were typically administered within one hour of each other, but not simultaneously in most cases.

**Figure 2:**
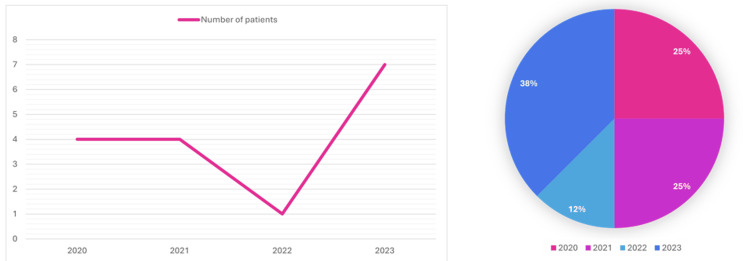
Trends and proportional distribution of CRE bacteremia cases at Tawam Hospital (2020–2023) Line graph: Displays annual CRE bacteremia cases, with stability in 2020–2021 (four cases/year), a sharp decline in 2022 (one case), and a significant rise in 2023 (seven cases). Pie chart: Represents proportional distribution by year, emphasizing 2023 (37.5%) as the year with the highest burden. Percentages for 2020 and 2021 are equal (25% each), while 2022 accounts for the smallest proportion (12.5%).

The distribution of CRE bacteremia cases at Tawam Hospital was analyzed over a four-year period (2020-2023). A composite figure (Figure 2) combines a line graph and a pie chart to illustrate both annual case counts and proportional distribution. The line graph reveals four cases in 2020 and 2021, followed by a sharp decline to one case in 2022 and a notable rise to seven cases in 2023, indicating a potential upward trend. The pie chart complements this by showing proportional distribution: 25% of total cases occurred in both 2020 and 2021, 12.5% in 2022, and 37.5% in 2023. This highlights the disproportionate increase in 2023, which accounted for over one-third of all cases, reinforcing concerns about rising incidence.

**Figure 3:**
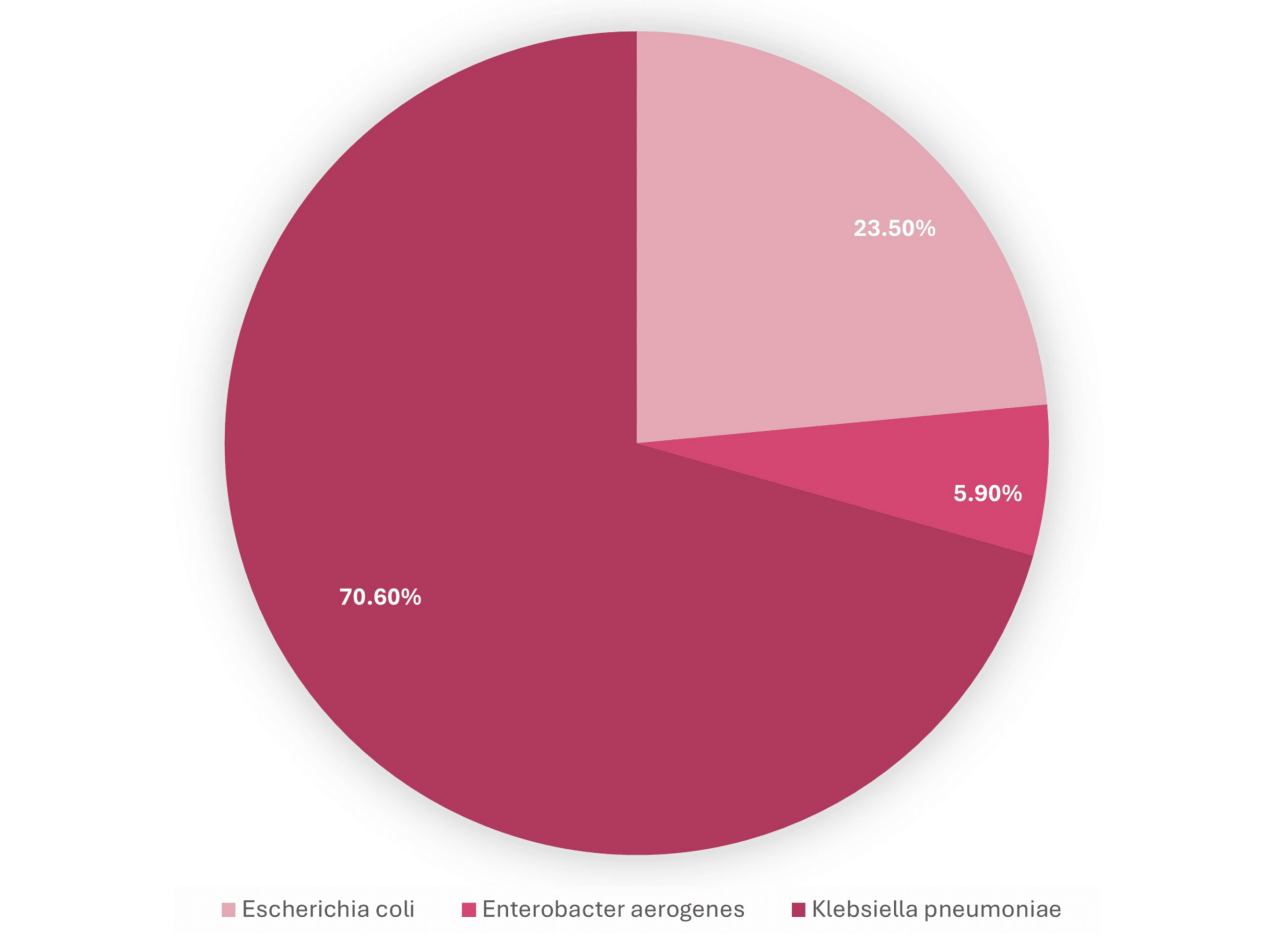
Distribution of Enterobacteriaceae species detected in carbapenem-resistant Enterobacterales (CRE) bacteremia cases at Tawam Hospital The pie chart illustrates the proportions of isolated *Enterobacteriaceae* species among patients with carbapenem-resistant Enterobacterales (CRE) bacteremia, with *Klebsiella pneumoniae* representing the majority of cases.

The distribution of *Enterobacteriaceae* species isolated from blood cultures is presented in a pie chart (Figure 3). *Klebsiella pneumoniae *was the most frequently identified organism, accounting for 70.6% of all isolates. *Escherichia coli* comprised 23.5% of cases, while *Enterobacter aerogenes* was identified in 5.9% of cases. This highlights *Klebsiella pneumoniae* as the predominant pathogen responsible for CRE bacteremia during the study period.

**Figure 4:**
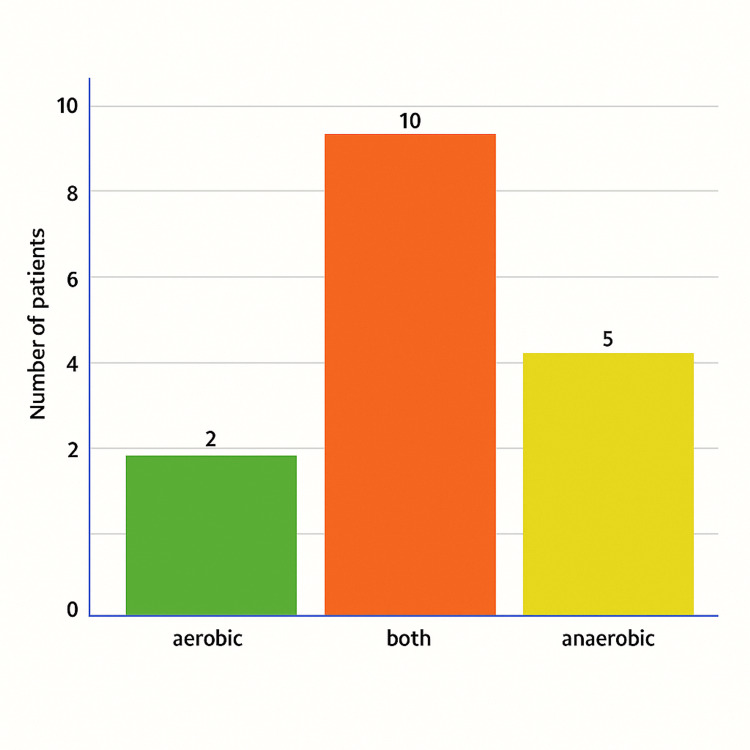
types of positive blood culture bottles detecting carbapenem-resistant Enterobacterales (CRE) bacteremia at Tawam Hospital The bar chart illustrates the distribution of blood culture bottle types that detected carbapenem-resistant Enterobacterales (CRE) bacteremia, showing a predominance of positivity in both aerobic and anaerobic bottles combined.

The types of blood culture bottles yielding positive results for CRE bacteremia were analyzed and are presented in a bar chart (Figure 4). In the majority of cases (58.8%), both aerobic and anaerobic culture bottles were positive. Aerobic bottles alone identified bacteremia in 11.8% of cases, while anaerobic bottles alone were positive in 29.4% of cases. This finding indicates that dual-bottle positivity was the most common pattern in detecting CRE bacteremia in this patient cohort.

**Figure 5:**
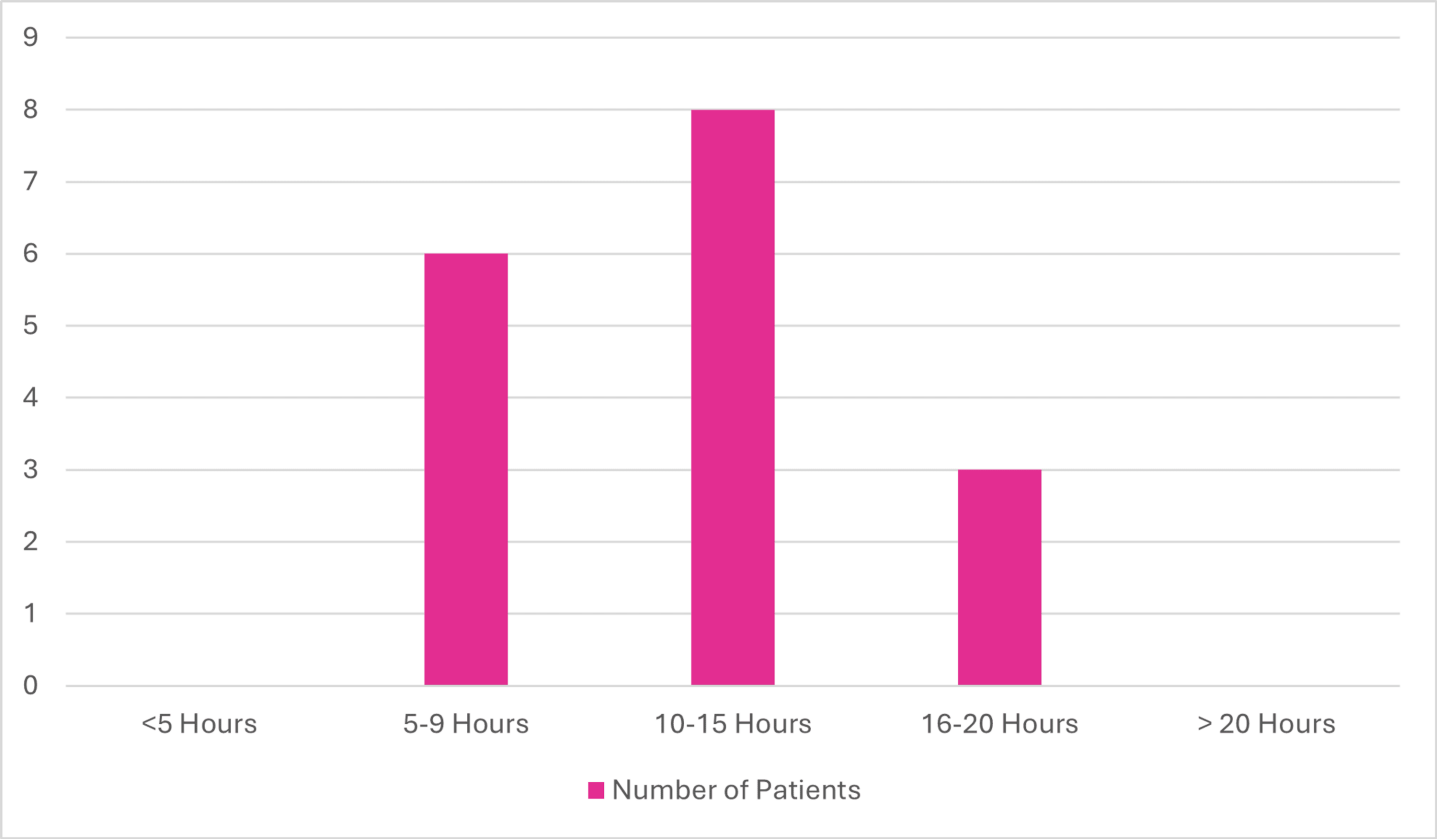
Time to detection of carbapenem-resistant Enterobacterales (CRE) bacteremia in blood cultures among hospitalized patients The bar chart shows the time intervals from blood culture collection to detection of carbapenem-resistant Enterobacterales (CRE) bacteremia, with the majority of cases detected between five and 15 hours. No correlation was observed between detection time and clinical outcomes.

The time to detection of bacteremia from blood cultures was evaluated among the study population and is summarized in a bar chart (Figure 5). No cases were detected in under five hours. Six patients (35.3%) had positive cultures detected between five and nine hours, eight patients (47%) between 10 and 15 hours, and three patients (17.6%) between 16 and 20 hours. No detections occurred after twenty hours. Further analysis showed no clear correlation between the time to detection and either disease severity or patient mortality.

Figure 5 illustrates the time from the onset of symptoms to the initiation of targeted therapy. A substantial delay (median: four days) was observed across the cohort. This delay may have contributed to adverse outcomes, particularly in immunocompromised patients, and highlights the need for earlier CRE detection and initiation of effective treatment.

The average time to receive antimicrobial sensitivity results was day five, with 15 cases (88%) having results released on day five, one case on day six, and one case on day seven after blood culture collection. The time to initiation of targeted therapy with CAZ-AVI and ATM showed some variability: 47% of patients started therapy by day four, 35% by day five, with isolated cases starting on day two, day six, and day eight.

**Figure 6:**
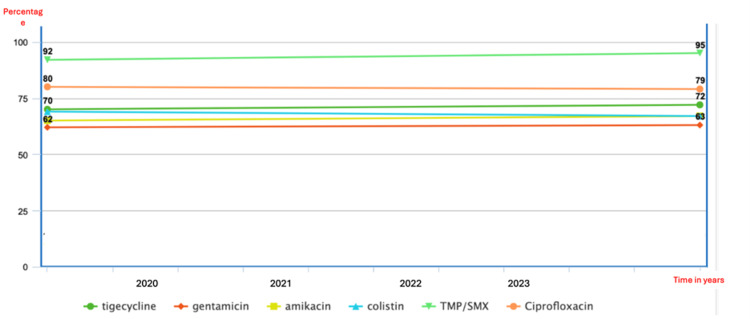
Resistance patterns (%R) of non-beta-lactam antibiotics among carbapenem-resistant Enterobacterales (CRE) bacteremia isolates at Tawam Hospital (2020–2023) The bar chart summarizes resistance rates to non-beta-lactam antibiotics, demonstrating consistently high resistance to ciprofloxacin, tigecycline, and trimethoprim-sulfamethoxazole (TMP/SMX) and comparatively lower resistance to amikacin.

Resistance patterns among non-beta-lactam antibiotics used for CRE bacteremia management were evaluated and are summarized in a bar chart (Figure 6). Ciprofloxacin and tigecycline exhibited consistently high resistance rates, exceeding 80% across the study period and reaching up to 95% in later years. Resistance to colistin and trimethoprim-sulfamethoxazole (TMP/SMX) was also substantial, beginning at approximately 60-68% and stabilizing around 70%. Gentamicin resistance was lower compared to amikacin, indicating some retained susceptibility within the aminoglycoside class. TMP/SMX resistance remained high throughout, starting at 84% and maintaining levels near 85%. These findings highlight the limited effectiveness of several non-beta-lactam agents against CRE isolates at our institution.

**Figure 7:**
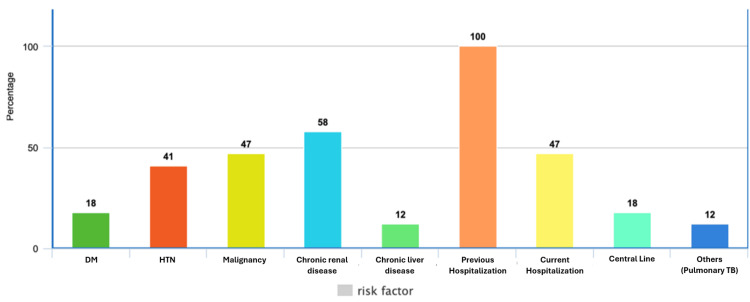
Distribution of clinical risk factors percentage among patients with carbapenem-resistant Enterobacterales (CRE) bacteremia at Tawam Hospital The bar chart illustrates the prevalence of major clinical risk factors among CRE bacteremia patients, with previous hospitalization or recent antibiotic use identified in all cases. X-axis abbreviations: DM = diabetes mellitus; HTN = hypertension; Malignancy = hematological/non-hematological; Chronic renal disease = structural or functional disorders; Chronic liver disease = hepatic conditions; Previous hospitalization = prior hospitalization or antibiotic administration within 90 days; Central line = PICC line, Port-a-Cath, etc.; Others = pulmonary tuberculosis or other conditions

The distribution of clinical risk factors among patients with CRE bacteremia is presented in a bar chart (Figure 7). The most common risk factor identified was a history of previous hospitalization or antibiotic exposure within the preceding 90 days, present in 100% of cases. Chronic renal disease was observed in 58% of patients, malignancy in 47%, and hypertension in 41%. Diabetes mellitus and central line usage were each present in 18% of patients, while chronic liver disease and other comorbid conditions, such as pulmonary tuberculosis, were observed in 12% of cases. In addition, 47% of patients developed bacteremia after 48 hours of hospitalization, indicating a nosocomial source. These findings highlight the significant burden of comorbidities and healthcare-associated exposures among patients affected by CRE bacteremia.

The demographic and clinical characteristics of patients with CRE bacteremia at presentation to the emergency department are summarized in Table 2. The table highlights individual patient data, including age, gender, presence of key comorbidities such as DM, HTN, CKD, and malignancy. It also outlines the primary clinical presentation and the identified septic focus for each case. Fever was the most frequent presenting symptom, often associated with underlying infections such as central line-associated bloodstream infections (CLABSI), pneumonia, complicated urinary tract infections (cUTI), or spontaneous bacterial peritonitis (SBP). Several cases involved polymicrobial sepsis and complex infectious sources, particularly among patients with malignancy or chronic renal disease.

**Table 2:** Summary of clinical risk factors, presentation, and identified septic focus in patients with carbapenem-resistant Enterobacterales (CRE) bacteremia at Tawam Hospital This table summarizes individual patient data, including age, gender, presence of diabetes mellitus (DM), hypertension (HTN), chronic kidney disease (CKD), and malignancy, along with clinical presentation at emergency department arrival and the identified infectious source. Abbreviations: DM = diabetes mellitus; HTN = hypertension; CKD = chronic kidney disease; HLH = hemophagocytic lymphohistiocytosis; BMT = bone marrow transplant; BPH = benign prostatic hyperplasia; cUTI = complicated urinary tract infection; SBP = spontaneous bacterial peritonitis; CLABSI = central line-associated bloodstream infection; S/P = status post; AML = acute myeloid leukemia

Year	Age	Gender	DM	HTN	CKD	Malignancy	Clinical presentation	Septic focus
2020	43	F	N	N	N	HLH, B-lymphoblastic leukemia/lymphoma, neutropenic	Fever, confusion	CLABSI, febrile neutropenia
2020	17	F	N	N	N	Failed BMT, progressive/refractory pancytopenia	Fever, cough	Pneumonia (CXR B/L infiltrates)
2020	75	M	N	Y	Y (Stage 2)	N	Leg wound discharge	Wet gangrene of left foot and right 5th toe, PVD
2020	81	M	Y	Y	Y (Stage 3)	N	Abdominal pain, pus in urine	CaUTI (History of left hydronephrosis, BPH)
2021	56	F	N	N	N	Cervical cancer with bilateral pleural effusion (diagnosed 1 month prior, not on treatment)	Vomiting, fatigue	Pneumonia (CXR B/L infiltrates)
2021	47	M	N	N	N	Large B-cell lymphoma, received R-CHOP (febrile neutropenia)	Cough, fever	Febrile neutropenia, left-sided pneumonia
2021	43	M	N	N	N	Recurrent metastatic bladder cancer, bilateral JJ stents, left nephrostomy tube	Flank pain, fever, turbid urine, dysuria	cUTI
2021	71	M	N	N	N	Bladder cancer (high-grade papillary transitional cell carcinoma), obstructive uropathy, bilateral nephrostomy	Fever, dysuria	cUTI
2022	87	M	Y	Y	N	N	Gastrointestinal bleeding (GIB)	Likely GI translocation with GIB
2022	61	M	N	N	N	N	Abdominal distension, tense ascites, confusion	SBP
2023	49	M	N	N	N	N	Fever, abdominal pain	cUTI, status post cystoscopy JJ stent removal
2023	54	F	N	Y	Y (Stage 5 on HD)	N	Abdominal distension, tense ascites	Unclear
2023	65	M	Y	Y	N	N	Fever, cough	cUTI, pneumonia, polymicrobial sepsis (Candida glabrata, ESBL E. coli, Enterococcus faecalis, Staph hominis, CRE Klebsiella)
2023	69	F	N	Y	N	Lung mass (not worked up)	Fever, increased respiratory secretions	cUTI vs pneumonia (underlying lung mass)
2023	47	F	N	N	N	N	Fever, vaginal bleeding, increased respiratory secretions	Pneumonia (CXR left infiltrate) vs cUTI
2023	74	F	N	Y	N	N	Fever	Unclear source, polymicrobial gram-negative infection
2023	17	M	N	N	N	AML with febrile neutropenia	Fever	Febrile neutropenia, CLABSI

**Figure 8:**
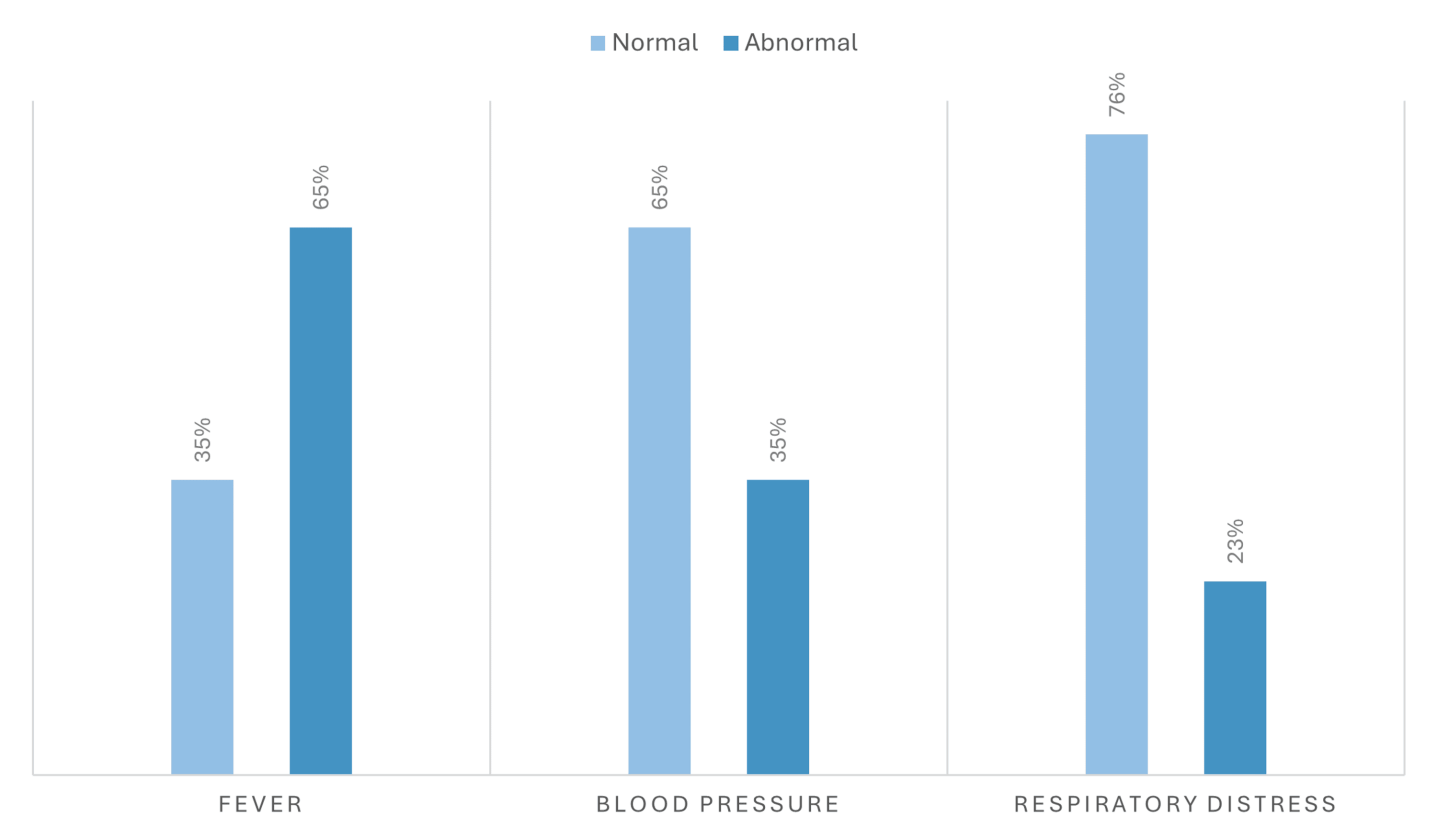
Clinical presentation profiles at diagnosis of carbapenem-resistant Enterobacterales (CRE) bacteremia A grouped bar chart illustrating three clinical parameters at presentation: Fever (≥38.0°C): 65% of patients presented with fever, 35% with normal temperature. Hypotension (systolic BP <90 mmHg): 35% of patients had hypotension, 65% maintained normal blood pressure. Respiratory distress (respiratory rate >22 breaths/min): 23% exhibited respiratory distress, 76% had normal respiratory rates.

In general, in this study, clinical presentation assessment was defined through the following measures (Figure 8). The presence of fever among patients with CRE bacteremia at presentation was evaluated. Fever, defined as a temperature ≥38.0°C, was identified in 65% of patients, while 35% of patients had normal temperature readings. This finding underscores the frequent association of systemic febrile response with CRE bloodstream infections. Blood pressure at presentation was analyzed as a marker of disease severity. Hypotension, defined as a systolic blood pressure <90 mmHg, was present in 35% of patients, while 65% maintained normal systolic blood pressure levels. This highlights the hemodynamic instability present in a substantial proportion of patients with CRE bacteremia. Respiratory status at admission was also assessed among patients with CRE bacteremia. Respiratory distress, defined as a respiratory rate exceeding 22 breaths per minute, was observed in 23% of patients, whereas 76% exhibited normal respiratory rates. This reflects the presence of respiratory compromise in a subset of cases at presentation.

**Figure 9:**
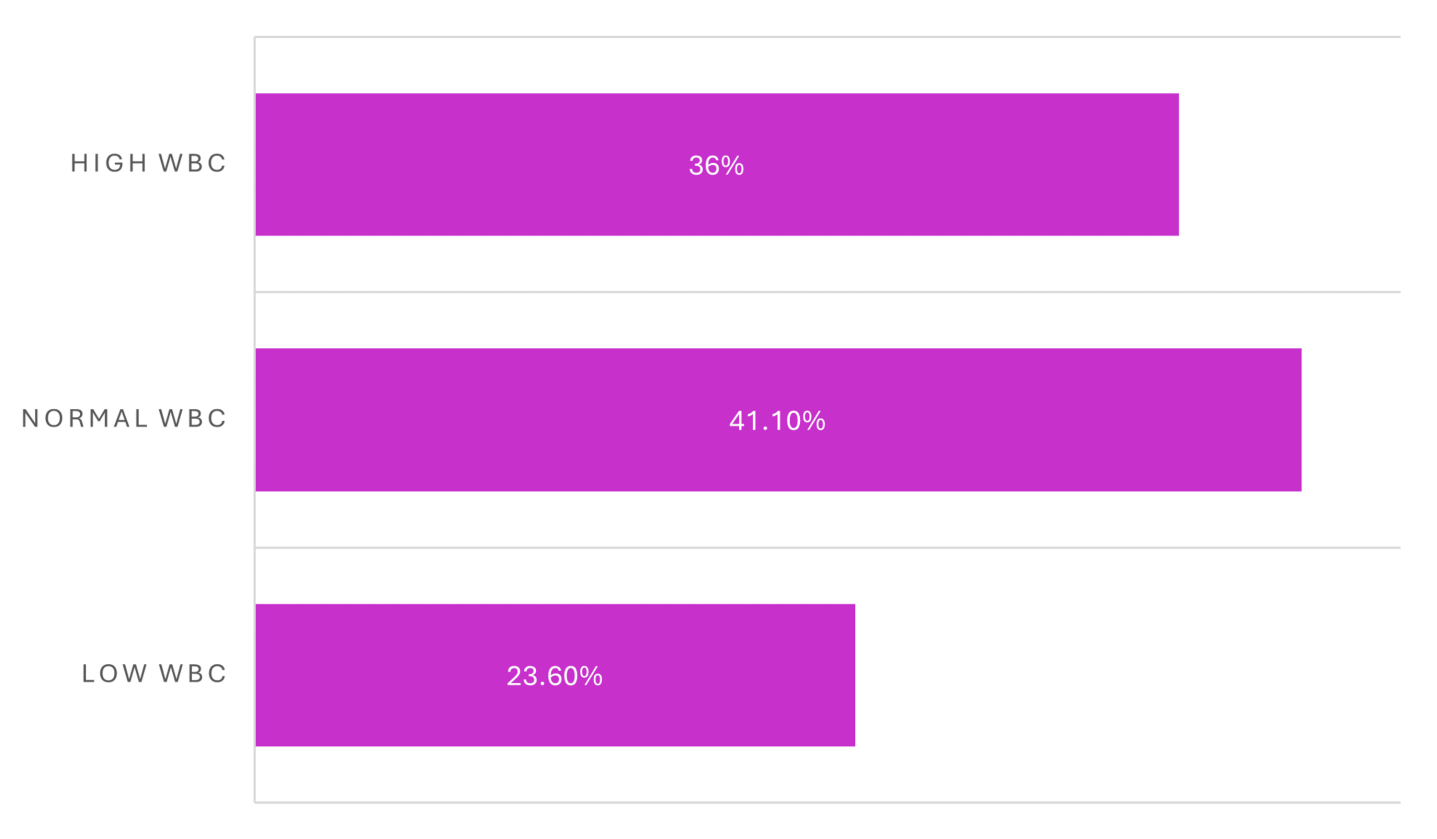
Distribution of white blood cell counts among patients with carbapenem-resistant Enterobacterales (CRE) bacteremia The bar chart illustrates the proportion of patients with low, normal, and high white blood cell counts at the time of diagnosis of carbapenem-resistant Enterobacterales (CRE) bacteremia.

The distribution of white blood cell counts among patients with CRE bacteremia is shown in Figure 9. Low WBC counts were observed in 23.6% of patients, while 41.1% had normal WBC counts at presentation. Elevated WBC counts were documented in 35.3% of cases. These findings demonstrate the variability of the systemic inflammatory response among patients with CRE bloodstream infections, with both leukocytosis and leukopenia observed in a substantial proportion of cases.

**Figure 10:**
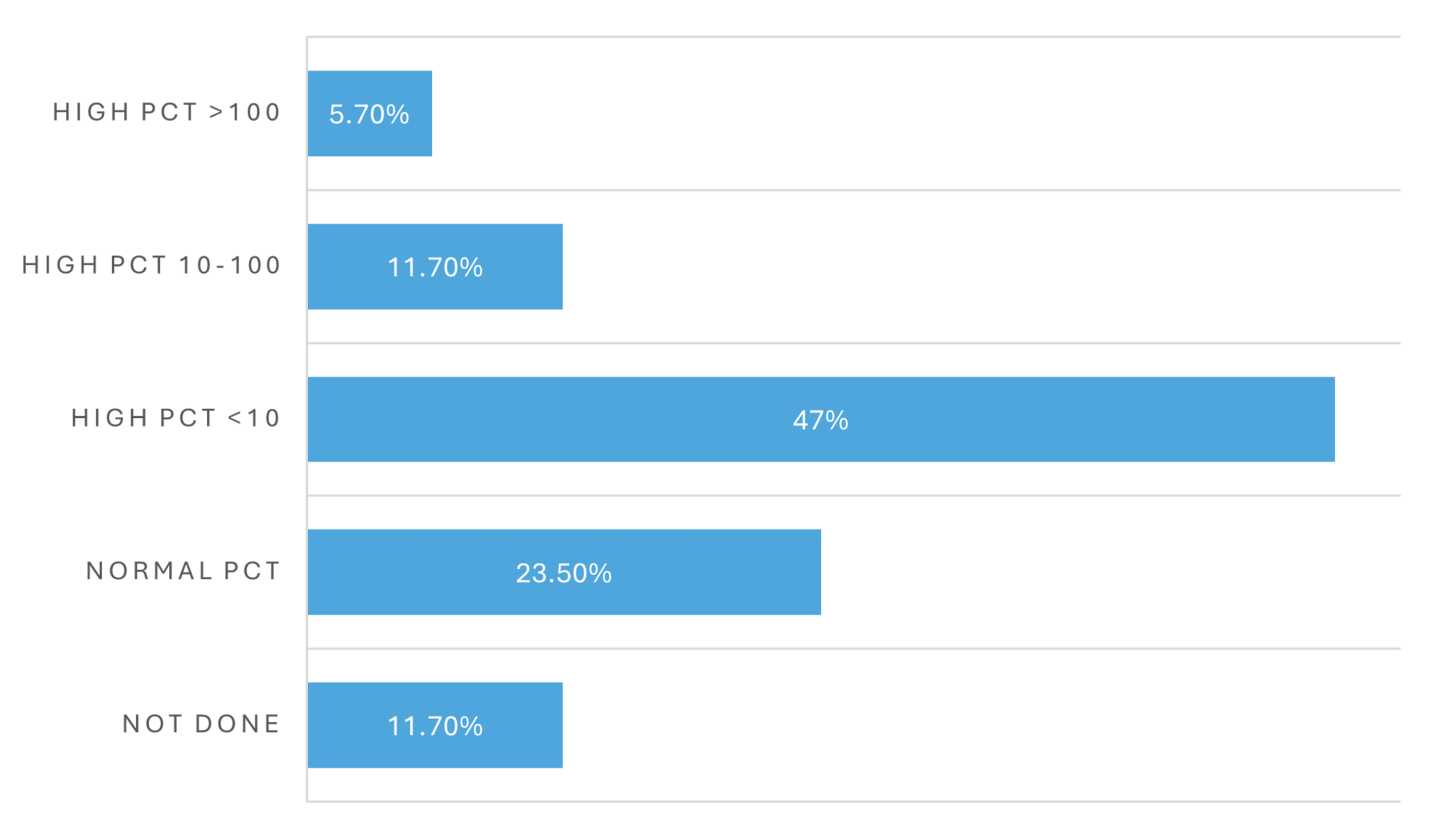
Distribution of procalcitonin (PCT) levels among patients with carbapenem-resistant Enterobacterales (CRE) bacteremia The bar chart shows the distribution of procalcitonin (PCT) levels at diagnosis, highlighting that the majority of patients had either normal or moderately elevated PCT levels. No significant association was found between PCT elevation and clinical outcomes.

Procalcitonin (PCT) levels were reviewed as an additional inflammatory marker among patients with CRE bacteremia and are summarized in Figure 10. PCT testing was not performed in 11.7% of cases. Normal PCT levels were identified in 23.5% of patients. Elevated PCT levels of less than 10 ng/mL were observed in 47% of patients, while levels between 10 and 100 ng/mL were seen in 11.7%. A markedly elevated PCT level exceeding 100 ng/mL was noted in 5.7% of patients. Further analysis indicated that there was no clear correlation between PCT levels and disease severity or mortality. 

Among the 17 cases of CRE bacteremia analyzed, five patients (29.4%) had concomitant polymicrobial bloodstream infections during the same hospitalization. The additional organisms isolated included *Enterococcus faecalis* in one case, *Enterococcus faecium* in another, and ESBL-producing *Escherichia coli* in a third case. In a fourth case, simultaneous blood cultures grew ESBL-producing *Proteus*, ESBL-producing *Klebsiella*, and *Pseudomonas* species. The fifth and most complex case involved multiple strains of carbapenem-resistant *Escherichia coli* and *Klebsiella* isolated from blood cultures. The first strain showed sensitivity to aztreonam in the presence of CAZ-AVI, the second strain exhibited intermediate susceptibility to aztreonam, cefiderocol, and colistin but retained sensitivity to tigecycline, and the third strain demonstrated resistance to aztreonam even with CAZ-AVI, intermediate susceptibility to cefiderocol, and sensitivity to amikacin and gentamicin. Unfortunately, the patient with these multiple resistant strains succumbed during the same hospitalization. These findings highlight the complexity and treatment challenges associated with polymicrobial and multidrug-resistant infections.

**Figure 11:**
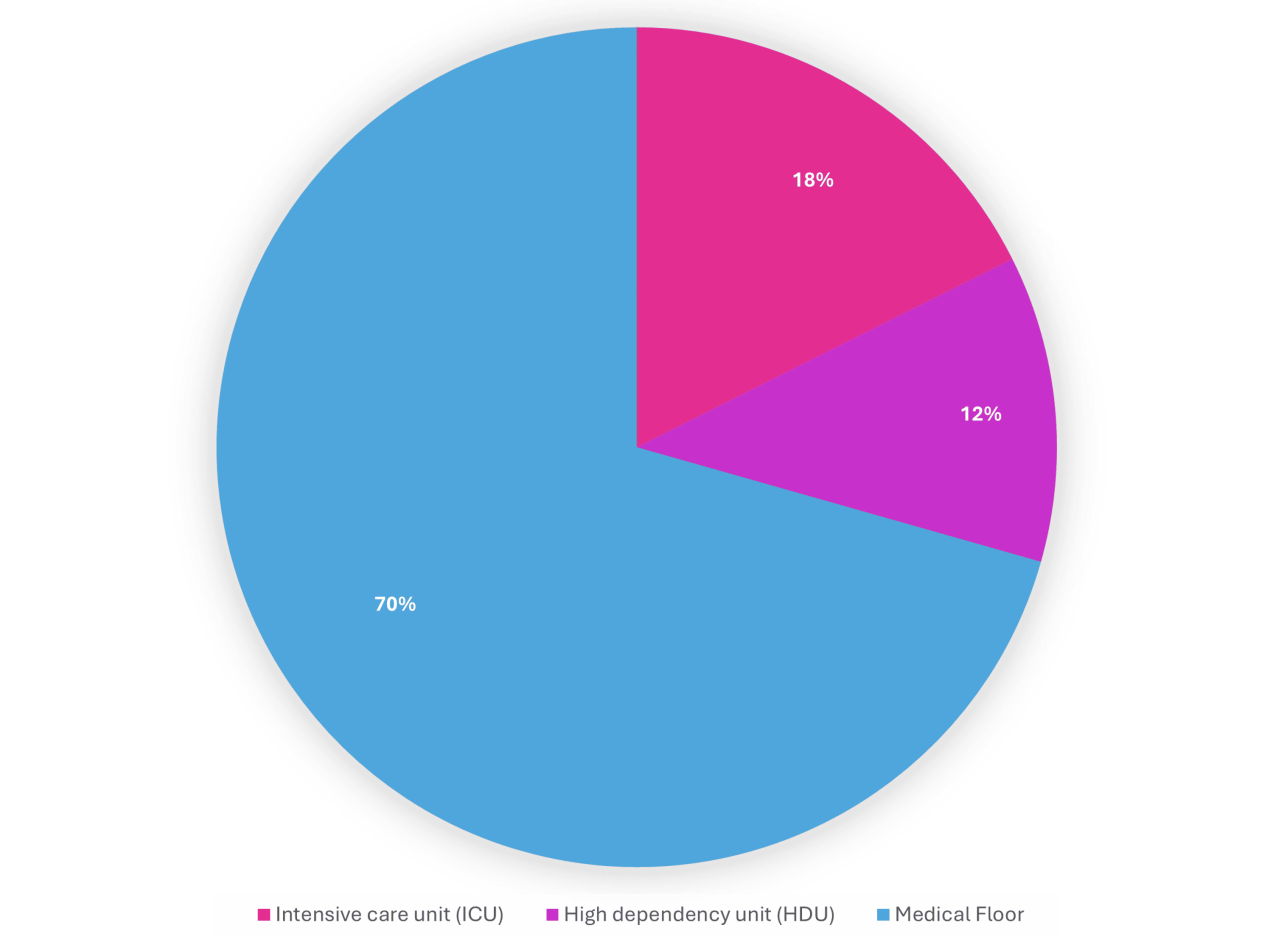
Level of hospital care among patients with Carbapenem-resistant Enterobacterales (CRE) bacteremia at Tawam Hospital The bar chart presents the distribution of patients according to hospital care levels, including ICU, high dependency unit (HDU), and general medical floor admissions during carbapenem-resistant Enterobacterales (CRE) bacteremia management.

The level of care required by patients with CRE bacteremia during hospitalization was analyzed and is illustrated in Figure 11. Seventeen percent (17.6%) of patients required admission to the intensive care unit (ICU), reflecting significant disease severity. The majority of patients (70.6%) were managed on the general medical floor, while 11.8% required admission to the high dependency unit (HDU). These findings suggest that although a considerable number of cases were severe, most patients were initially managed outside of critical care settings.

**Figure 12:**
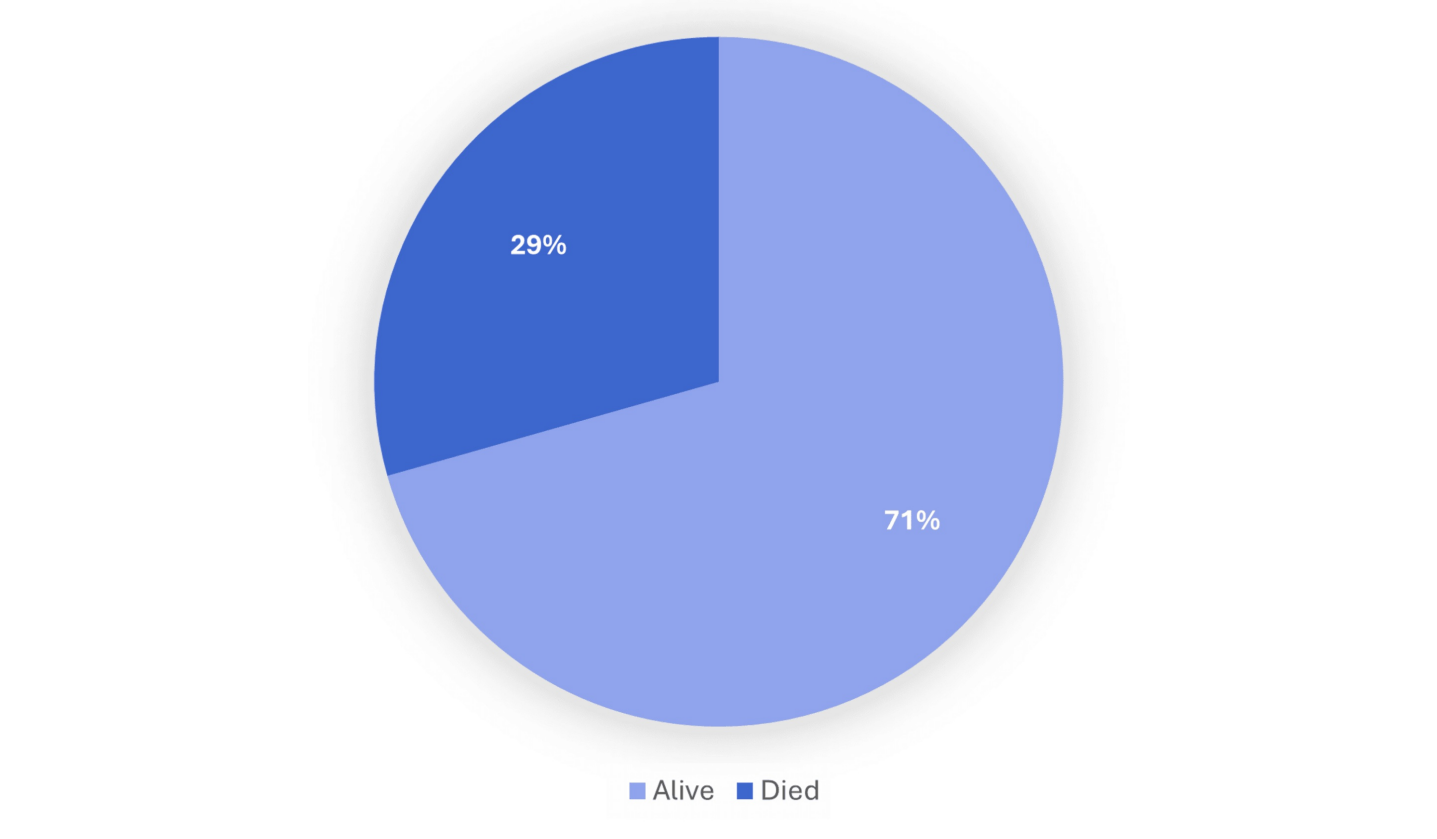
In-hospital mortality outcomes of patients with carbapenem-resistant Enterobacterales (CRE) bacteremia The bar chart illustrates survival outcomes among patients diagnosed with carbapenem-resistant Enterobacterales (CRE) bacteremia during the same hospitalization, showing that nearly one-third of patients did not survive the admission.

The in-hospital mortality rate among patients with CRE bacteremia was analyzed and is illustrated in Figure 12. During the same admission in which bacteremia was diagnosed, 29.4% of patients died, while 70.6% survived and were discharged. This finding highlights the considerable mortality burden associated with CRE bloodstream infections despite targeted combination antimicrobial therapy.

**Figure 13:**
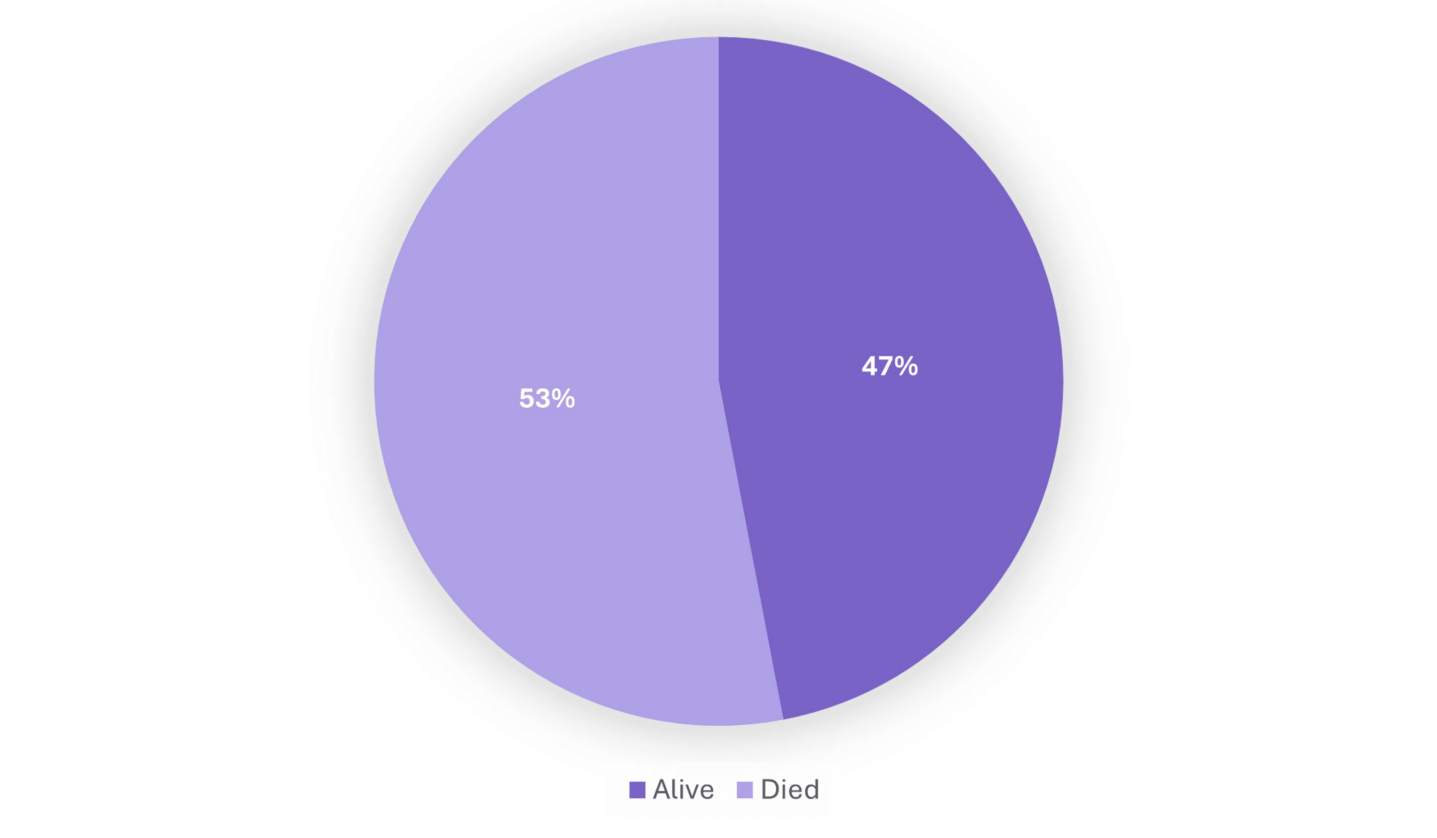
Overall four-year mortality outcomes in patients with carbapenem-resistant Enterobacterales (CRE) bacteremia The bar chart demonstrates overall survival rates among patients with carbapenem-resistant Enterobacterales (CRE) bacteremia after four years of follow-up, with a mortality rate exceeding 50%.

Long-term outcomes for patients with CRE bacteremia were assessed over a four-year follow-up period and are illustrated in Figure 13. At the end of follow-up, 53.0% of patients had died, while 47.0% were alive. These results reflect the high long-term mortality burden associated with CRE infections, further emphasizing the severity and lasting impact of these resistant pathogens.

The length of hospital stay associated with admissions for CRE bacteremia was evaluated. The median length of stay among the studied cohort was 19.0 days. This reflects the significant healthcare burden posed by CRE infections, often requiring prolonged hospitalization for management of severe infections, administration of targeted antimicrobial therapy, and addressing associated complications.

## Discussion

CRE infections remain a significant therapeutic challenge, particularly in patients with bloodstream infections. Although novel antibiotic combinations have expanded treatment options for resistance mediated by serine-carbapenemases, infections caused by metallo-beta-lactamase (MBL)-producing organisms continue to be associated with limited effective therapies and high mortality rates [9,10].

In our study, the absence of molecular genotyping for carbapenemase enzymes necessitated reliance on microbiological methods to determine antimicrobial susceptibility and evaluate synergy between CAZ-AVI and ATM. Although four in vitro testing methods exist for evaluating CAZ-AVI and ATM synergy (broth disk elution, disk stacking, strip stacking, and strip crossing), our facility employed a modified E-test disc diffusion method, which previous validation studies have shown to exhibit high sensitivity and specificity (92% and 88%, respectively) [10]. This method offered a practical and reproducible approach for synergy assessment in the absence of genetic testing capabilities.

The antimicrobial dosing regimens followed pharmacokinetic recommendations, including renal dose adjustments [11]. Importantly, CAZ-AVI and ATM were administered within a one-hour window rather than simultaneously, reflecting real-world bedside practices and pragmatic care adjustments [2].

An increasing trend in CRE bacteremia cases was observed over the study period, particularly during 2023. This may reflect either a genuine rise in incidence, shifts in hospital epidemiology, or improved detection practices [2]. *Klebsiella pneumoniae *was the predominant organism isolated, accounting for more than two-thirds of cases, a finding that aligns with both local UAE surveillance data [12] and global epidemiological trends [13].

The predominance of *K. pneumoniae *may be linked to its propensity to acquire mobile genetic elements encoding carbapenemases, a phenomenon exacerbated by antibiotic overuse and horizontal gene transfer in healthcare settings [14]. Regional antibiotic stewardship initiatives, such as the UAE National AMR Action Plan [15], could mitigate this trend by optimizing prescribing practices.

Despite the availability of combination antimicrobial therapy, patient outcomes remained poor. The in-hospital mortality rate during the index admission was 29.4%, and overall four-year mortality reached 53.0%. These figures reinforce the aggressive nature of CRE bloodstream infections and the complex interplay of host factors influencing outcomes [16]. Identified risk factors in our cohort, such as prior hospitalization, chronic kidney disease, hypertension, and malignancy, were consistent with those previously reported to predict adverse outcomes in similar patient populations [17,18].

Immunosuppression, a key risk factor in our study, likely impairs pathogen clearance and exacerbates drug resistance through subtherapeutic antibiotic penetration in tissues [19]. This aligns with global data showing higher mortality rates in immunocompromised patients with CRE bacteremia [20].

Laboratory findings revealed considerable heterogeneity in inflammatory markers. While white blood cell (WBC) counts varied among patients, PCT levels did not correlate clearly with either disease severity or mortality, limiting their predictive value in this setting. Although PCT is frequently elevated in bacterial infections, its prognostic utility in CRE bacteremia, particularly among severely immunocompromised patients, appears limited based on our data [21].

Polymicrobial bloodstream infections were noted in approximately 29.0% of cases. In particular, one highly complex case involved multiple strains of CRE with divergent resistance patterns, ultimately culminating in treatment failure and mortality. Such polymicrobial infections, especially with multidrug-resistant organisms, present extraordinary therapeutic challenges and are associated with poor clinical outcomes [22,23].

Our findings suggest that while the combination of CAZ-AVI and ATM represents a promising therapeutic approach against MBL-producing CRE, patient outcomes remain heavily dependent on host comorbidities, source control, and the timely initiation of effective therapy [10]. Importantly, in settings where access to molecular diagnostic tools is limited, our results demonstrate the feasibility of using phenotypic synergy testing to guide treatment decisions. This has direct implications for clinical practice in resource-constrained environments, where early empiric use of this combination - guided by clinical suspicion and local resistance trends - may improve outcomes. These findings support the need to integrate this regimen into treatment protocols for suspected CRE bacteremia and emphasize the importance of investing in diagnostic capacity to optimize antimicrobial selection and stewardship.

Delays in initiating effective therapy (see Figure 5) reflect common diagnostic and logistical hurdles in CRE management. Reducing these delays through faster laboratory turnaround and earlier empiric coverage may help improve survival rates. The lack of PCT correlation with outcomes likely reflects impaired inflammatory responses in immunocompromised patients, limiting the biomarker's utility in this population. This further supports the need for clinical judgment and multi-parameter assessment in managing CRE infections.

While the overall mortality in our cohort was high, it is important to distinguish between deaths directly attributable to CRE bacteremia and those primarily related to underlying disease processes. Many patients had significant comorbid conditions-including hematologic malignancies, advanced chronic kidney disease, and neutropenia, which likely contributed to poor outcomes. In several cases, CRE bacteremia may have acted as a terminal event in the setting of progressive, irreversible illness. This overlap makes it difficult to definitively assign the cause of death, but underscores the importance of early detection, source control, and individualized treatment in high-risk patients.

Limitations

This study has several limitations. First, the retrospective design and absence of a control group limit the ability to infer causality or compare treatment efficacy across regimens. Second, the small sample size (n = 17) and single-center setting restrict the generalizability of the findings. Third, the absence of molecular carbapenemase typing precluded precise classification of resistance mechanisms, which may have influenced treatment response and clinical outcomes [13]. In addition, the sample size did not allow for multivariate analysis, reducing the statistical strength of some associations. Despite these constraints, the study provides valuable real-world insights into the use of CAZ-AVI and ATM in managing CRE bacteremia in a region with limited published data.

## Conclusions

This study underscores the growing challenge of CRE bacteremia in the UAE, reflecting a broader global threat driven by rising antimicrobial resistance. While limited by a small sample size, the findings suggest that healthcare-associated exposures, immunocompromised states, and comorbidities may contribute to adverse outcomes in affected patients. The use of CAZ-AVI and ATM combination therapy may offer clinical benefit in select cases, although mortality remains high in vulnerable populations. These results highlight the importance of antimicrobial stewardship, access to advanced diagnostics, and strict infection control practices in managing CRE. To further validate and expand upon these findings, future research should include multicenter cohorts and incorporate molecular resistance profiling to better guide targeted therapies and public health strategies.
